# Contactless quasi-steady-state photoconductance (QSSPC) characterization of metal halide perovskite thin films

**DOI:** 10.1038/s41598-023-37745-2

**Published:** 2023-07-10

**Authors:** Benjamin Grimm, Sascha J. Wolter, Jan Schmidt

**Affiliations:** 1 Institute for Solar Energy Research Hamelin (ISFH), Am Ohrberg 1, 31860 Emmerthal, Germany; 2 Department of Solar Energy, Institute of Solid-State Physics, Leibniz University Hannover, Appelstr. 2, 30167 Hannover, Germany

**Keywords:** Characterization and analytical techniques, Solar cells

## Abstract

We apply the contactless quasi-steady-state photoconductance (QSSPC) method to co-evaporated methyl ammonium lead iodide (MAPbI_3_) perovskite thin-films. Using an adapted calibration for ultralow photoconductances, we extract the injection-dependent carrier lifetime of the MAPbI_3_ layer. The lifetime is found to be limited by radiative recombination at the high injection densities applied during the QSSPC measurement, enabling the extraction of the electron and hole mobility sum in the MAPbI_3_ using the known coefficient of radiative recombination of MAPbI_3_. Combining the QSSPC measurement with transient photoluminescence measurements, performed at much lower injection densities, we obtain the injection-dependent lifetime curve over several orders of magnitude. From the resulting lifetime curve, we determine the achievable open-circuit voltage of the examined MAPbI_3_ layer.

## Introduction

Metal halide perovskites like methyl ammonium lead iodide (MAPbI_3_) have emerged over the past decade as a new and promising class of materials for the application in low-cost, high-efficiency solar cells^1^. With MAPbI_3_ being one of the first compositions within the class of metal halide perovskites, it is also the most studied so far^1,2^. It piqued the interest of the photovoltaic community with fast-growing power conversion efficiencies (PCEs) enabled by a high absorption coefficient, a direct bandgap of 1.6 eV, relatively high charge carrier mobilities and long charge carrier lifetimes. Of these crucial material parameters the charge carrier lifetime in particular directly affects the efficiency of perovskite-based solar cells because of its strong dependence on the composition, the manufacturing method and the degree of contamination of the perovskite layer. Accurate measurements of the carrier lifetime are hence of utmost importance in perovskite research. Frequently neglected in perovskite research is, however, the fact that the carrier lifetime is not a constant value, but depends on the excess carrier concentration, i.e. the injection level, within the layer. Unfortunately, the lifetime measurement techniques predominantly applied in perovskite research, such as the time-resolved photoluminescence (TRPL) method^3–5^, measure the carrier lifetimes using dynamic approaches without determining the exact excess carrier concentrations present during the measurements. In this paper, we employ the contactless quasi-steady-state (QSSPC) method, a measurement technique developed to measure the injection-dependent lifetime in silicon by inductively coupling an illuminated sample to an rf bridge while simultaneously recording the time-dependent illumination intensity^6^. We apply the method, as used before only for silicon characterization, for the first time to metal halide perovskite layers and demonstrate that lifetimes as well as excess carrier concentrations can be extracted from the measurements at the same time. In order to expand the injection-range of the injection-dependent lifetime curves, we combine QSSPC with TRPL measurements and deduce suns-implied voltage characteristics from it.

## Experimental

The quasi-steady-state photoconductance (QSSPC) measurement technique is a standard tool in silicon-based photovoltaics, where it is routinely used for injection-dependent measurements of the carrier lifetime of silicon wafers. It is based on inductive coupling of the semiconductor sample via a coil to an rf bridge circuit, the output voltage of which depends linearly on the photoconductance of the measured sample. The methodology was introduced by Sinton and Cuevas in 1996^6^ and evolved during the past decades into a mighty contactless and easy-to-apply tool for the characterization of bulk and surface recombination losses in silicon wafers and non-metallized solar cell precursors. In this contribution, we apply the QSSPC method, as used before only for silicon characterization, for the first time to metal halide perovskite layers.

**Figure 1:**
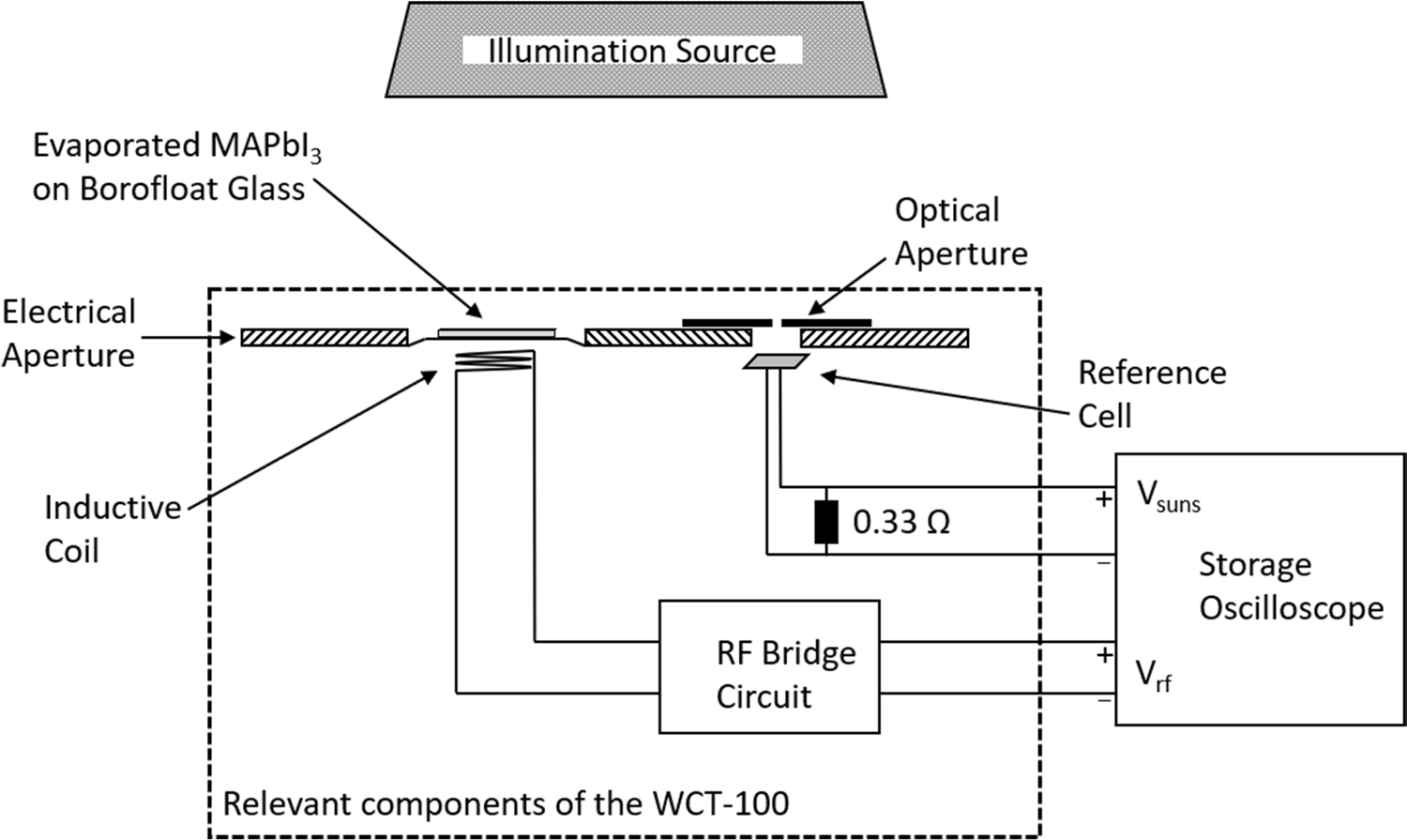
Schematic diagram of the WCT-100 system with a MAPbI_3_ perovskite sample placed on top of the coil.

Figure 1 shows a sketch of the WCT-100 system (Sinton Instruments) used in this study. The sample, which is normally a silicon wafer, is placed above the coil, which inductively couples the sample to an rf bridge circuit. The output voltage of the rf bridge circuit *V*_rf_ depends linearly on the conductance of the sample, which changes due to illumination with a flash installed above the sample. Before a measurement under illumination is recorded, the circuit is balanced by an adjustable capacitor and resistance (not shown in Fig. 1), so that the output bridge voltage *V*_rf_ is set at 100 ± 10 mV. During the illumination by the flash, a calibrated reference solar cell measures the intensity as a function of time. The reference cell is kept close to short-circuit conditions by means of a 0.33 Ω resistor connected in parallel to the reference solar cell, so that the output voltage *V*_suns_ can be directly translated into the illumination intensity *I* in suns, which is recorded by a two-channel storage oscilloscope as a function of time. From this signal, the photogeneration rate *G(t)* in the silicon sample is calculated. In parallel, on the other channel of the oscilloscope, the *V*_rf_ signal is recorded as a function of time *t*. A calibration of the rf bridge circuit is performed by using samples of different well-known conductances. Using this calibration curve, the photoconductance can be deduced from the *V*_rf_ signal. As the mobilities of silicon are well known, these are used to calculate the excess carrier concentration Δ*n* from the photoconductance signal. Hence, Δ*n*(*t*) is obtained directly form the *V*_rf_(*t*) signal. The flash decay time constant is adjusted in a way that it decays much slower compared to the excess carrier lifetime of the silicon sample under investigation. The sample can then be regarded as under “quasi-steady-state” conditions and the carrier lifetime τ can be easily calculated during the decaying flash by means of the equation τ = Δ*n*/*G*, where it is assumed that the recombination rate equals the generation rate, as it is characteristic for steady-state conditions, at every point in time. As Δ*n* decays during the decaying flash, one obtains an injection-dependent τ(Δ*n*) curve during only one flash. Due to its elegance and simplicity, the QSSPC measurement technique has evolved to a central characterization tool in silicon photovoltaics in the past two decades. Details of the Sinton measurement tool and on the detailed data evaluation procedure are reported in Ref.^7^.

In the present study, we position the glass substrate with a 500 nm MAPbI_3_ perovskite layer (sample preparation see Supplemental A) facing towards the flash light in the center of the 1.6 cm-diameter coil, which is embedded in epoxy resin. The flasher head is positioned in our measurement only approximately 4 cm above the sample, so it is much closer to the perovskite sample compared to a standard silicon wafer measurement, where distances of 30 to 65 cm are typical, leading to significantly higher light intensities in our measurements of perovskite samples compared to standard silicon wafer measurements. For the reference silicon solar cell, an optical aperture diameter of only 1.9 mm was chosen to allow the measurement of the much higher illumination intensities on the sample surface (40–590 suns) required to obtain a sufficiently high signal-to-noise ratio for the measurement of the perovskite thin-films. The output bridge voltage of Δ*V*_rf_ relative to the balanced voltage is the central measurement quantity, because it is directly related to the photoconductance of the examined perovskite sample. In the case of silicon wafers, the bridge voltage depends linearly on the silicon wafer conductance, which, however, cannot be necessarily expected for perovskite layers exhibiting much lower photoconductances due to the fact that they are two orders of magnitude thinner and have significantly lower carrier lifetimes than a typical silicon wafer. Despite the expectation that the perovskite signal would be many orders of magnitude lower than the silicon signal and maybe not even detectable at all, we were surprised to immediately observe a much more pronounced Δ*V*_rf_ signal than expected.

**Figure 2:**
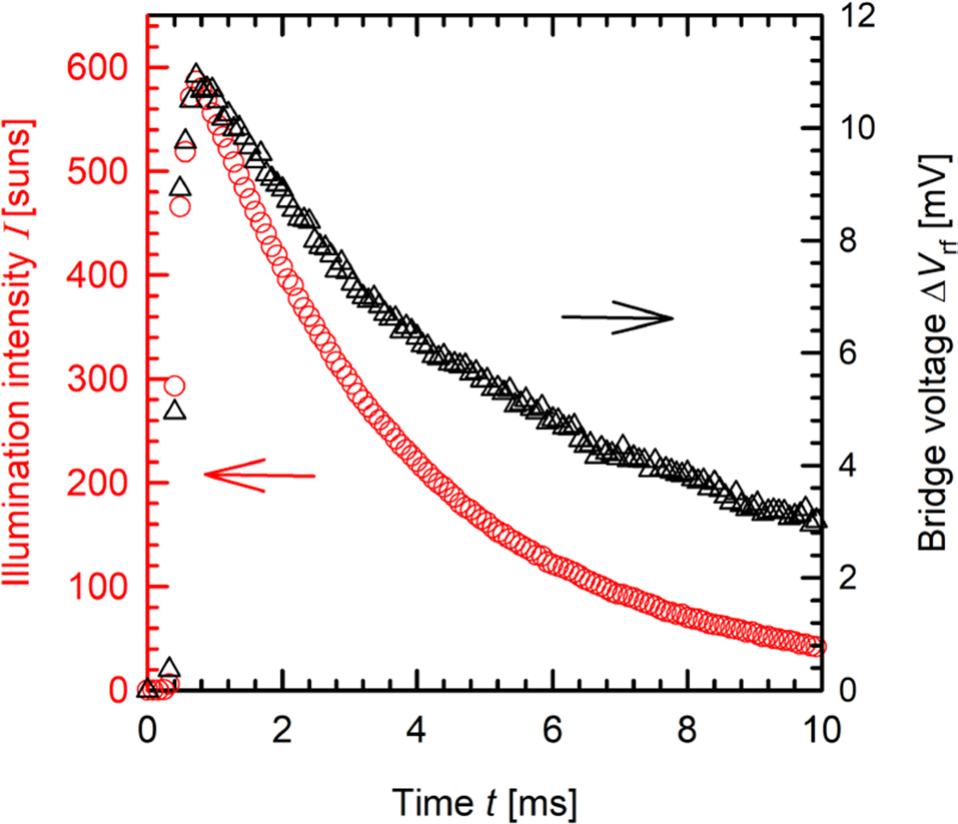
Exemplary quasi-steady-state photoconductance (QSSPC) measurement of a 500 nm thick MAPbI_3_ layer on borofloat glass using a Sinton WCT100 system. The flasher is located only 4 cm above the perovskite sample and the reference cell. A total number of five decay curves were averaged by the storage oscilloscope.

Figure 2 shows an exemplary measurement of a 500 nm thick MAPbI_3_ perovskite layer on a borofloat substrate using the Sinton WCT-100 tool during illumination of the sample with an exponentially decaying flash light pulse. A total number of five decay curves were averaged. The red circles show the intensity decay obtained from the reference cell and the black triangles show the output voltage Δ*V*_rf_ produced by the perovskite. At the peak illumination intensity of 590 suns, the output bridge voltage Δ*V*_rf_ is 11 mV. After 10 ms, the flash intensity decays to 40 suns and the Δ*V*_rf_ to 3 mV. Over the entire intensity range, the signal-to-noise ratio of the Δ*V*_rf_ measurement signal is sufficient to allow for a detailed analysis. In general, we find that for light intensities below ~ 40 suns, our current setup is not suitable to provide a sufficiently high Δ*V*_rf_ signal-to-noise ratio.

The unexpectedly large Δ*V*_rf_ signal measured for perovskite thin-films led to the suspicion that the sensitivity of the system is significantly higher for ultralow photoconductances. In order to verify this hypothesis, we have performed a thorough calibration of our WCT-100 system for ultralow conductances.

**Figure 3:**
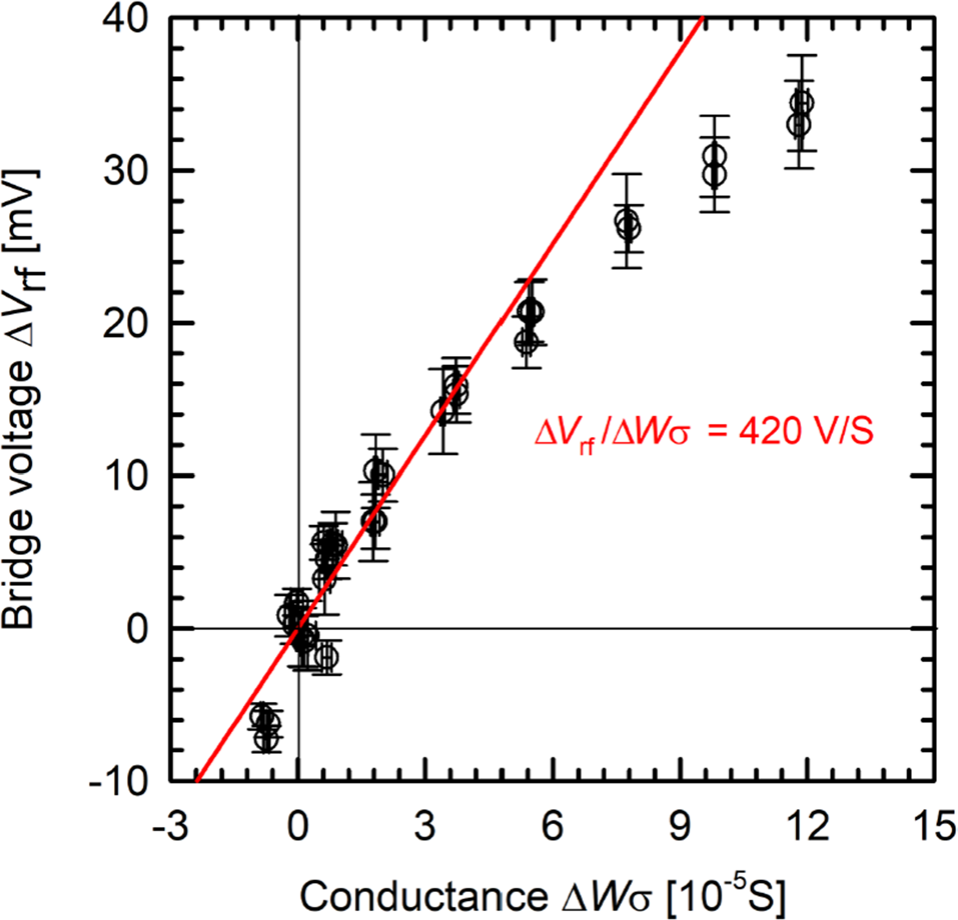
Calibration curve of the inductive coil for ultralow conductances using 200 $\Omega cm$ p-type silicon wafers of various thicknesses W. Wafer thicknesses were varied by means of etching. At Δ*V*_rf_ = 0 mV, the conductance ΔWσ was set at zero. The red line shows a proportional fit of Δ*V*_rf_ versus ΔWσ in the relevant voltage range of up to 11 mV.

For this calibration, we use 200 Ωcm *p*-type float-zone silicon wafers. The 6″ wafers are laser-cut into 2.5 × 2.5 cm^2^ samples and gradually etched thin using a 50% KOH etchant solution at 90 °C to reduce the thickness of initially 285 µm down to a minimum of 30 µm. Each calibration sample was individually measured by a thickness gauge at nine equally spaced spots to determine the respective average thickness. Before we measure the samples, we balance the rf-bridge once under air to a *V*_rf_ value of 100 ± 10 mV. Each calibration sample was then placed on top of the coil and the corresponding change in bridge voltage Δ*V*_rf_ was measured. A slight drift in the bridge voltage *V*_rf_ over time was compensated by rebalancing whenever *V*_rf_ left the 100 ± 10 mV range. Figure 3 shows the resulting calibration curve of Δ*V*_rf_ as a function of the wafer conductance Δ*Wσ*, as calculated from their known resistivity ρ = 1/σ and thicknesses *W*.

Note that we use the wafer conductance Δ*W*σ in Fig. 3, which is set at zero at a measured bridge voltage of Δ*V*_rf_ = 0. A proportional fit for the bridge voltage Δ*V*_rf_ as a function of Δ*W*σ in the typical perovskite measurement range of Δ*V*_rf_ up to 11 mV is shown as red line in Fig. 3. The resulting sensitivity of 420 V/S for the ultralow conductances of below 3 × 10^–5^ S for the perovskite thin films is one order of magnitude larger than the sensitivity of 50 V/S in the range where silicon samples are usually measured. Our calibration hence verifies the hypothesis that at ultralow conductance values, the WCT-100 setup is significantly more sensitive compared to the typical measurement range applied for silicon wafers. Hence, the system is suitable for measuring samples with very low photoconductance values, such as metal halide perovskite thin films. Note that, in good agreement with our findings reported here, a strongly increased sensitivity of the WCT-100 system for very low conductances has been reported before by McIntosh et al.^7^.

Applying our low-conductance calibration to the measurement shown in Fig. 2 provides the respective photoconductance and divided by the film thickness *d*, the photoconductivity Δσ, which can then be converted into the excess carrier concentration in the perovskite layer Δ*n* = Δσ/*q*µ_B_ using the mobility sum of electrons and holes µ_B_ = µ_n_ + µ_p_ and the elementary charge *q*. According to reports on high-quality MAPbI_3_ perovskite layers, µ_B_ is expected to be in the range between 10 and 20 cm^2^/Vs^2,8,9^.

Besides measuring the sample’s photoconductivity, the illumination intensity is simultaneously tracked by the calibrated solar cell. The generation rate *G* = *I* × *J*_sc_/*qd* is then calculated from the measured illumination intensity *I* in suns. We calculate the nominal short-circuit current density of the sample *J*_*sc*_ by integration of the tabulated AM1.5G Spectrum Φ(λ)^10^ multiplied by the measured absorption *A*(λ) of the MAPbI_3_ thin-film. It is important to note that there is no antireflection coating on our examined MAPbI_3_ thin-film. The calculated *J*_sc_ of 12.1 mA/cm^2^ is hence somewhat lower than in our solar cells processed on the same material.

The carrier lifetime τ_QSSPC_ of the perovskite layer is then calculated using the standard QSSPC equation τ_QSSPC_ = Δ*n*/*G*, resulting in an injection-dependent lifetime curve as in the case of the silicon wafer characterization.

## Results and discussion

**Figure 4:**
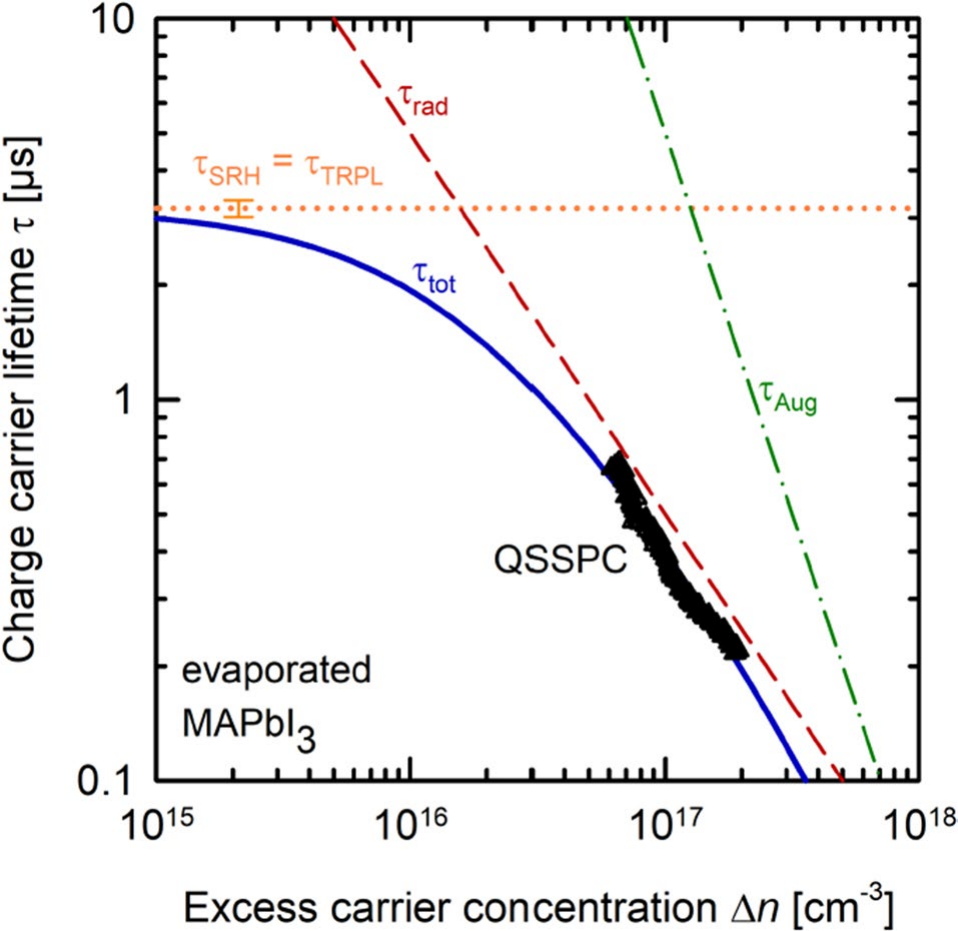
QSSPC measurement of the injection-dependent lifetime τ_QSSPC_(Δn) (triangles) of a MAPbI_3_-thin-film perovskite. An addition TRPL measurement of the same sample at low injection density (< 10^15^ cm^−3^) results in a lifetime of 3 µs, which is identified with the Shockley–Read–Hall (SRH) lifetime of the layer. τ_tot_ (solid blue line) is the calculated total lifetime, which is limited by the radiative lifetime τ_rad_ (red dashed line) in the injection range of the QSSPC measurement. Assuming a radiative recombination coefficient of k_rad_ = 2 × 10^−11^ cm^3^/s, as reported in the literature^5^, a mobility sum of µ_B_ = 17 cm^2^/Vs is extracted from the QSSPC measurement. For completeness, the Auger lifetime τ_Aug_ is also shown (green dash-dotted line).

Figure 4 shows the τ_QSSPC_(Δ*n*) curve (triangles) extracted from the QSSPC measurement of the evaporated MAPbI_3_ layer shown in Fig. 2. On the same sample, we have performed a TRPL measurement of the carrier lifetime, which is frequently used to measure carrier lifetimes in perovskite layers^2–4^. This measurement is dynamic and provides an absolute value when evaluating the asymptotic decay of the measured time-resolved PL signal (see Supplemental B). An exponential decay fit to the asymptotic decay results for the examined sample in a lifetime of 3.18 ± 0.16 µs, which is in line with other reported lifetimes of several microseconds measured in MAPbI_3_^11,12^. The exact injection density during the time resolved PL measurement is not known but is estimated to be  ≤ 10^15^ cm^−3^ and, hence, the measured TRPL lifetime τ_TRPL_ is expected to be limited by defect-related Shockley–Read–Hall (SRH) recombination as well as the surface recombination. For simplicity, we identify the measured TRPL lifetime with the SRH lifetime of the MAPbI_3_ layer τ_SRH_ = τ_TRPL_, which is assumed to be constant for the injection range shown in Fig. 4 (orange dotted line). Note that the SRH lifetime also includes the surface recombination, which is, however, very low in our sample. Also included in Fig. 4 are the calculated injection-dependent radiative lifetime τ_rad_ (red dashed line) and the Auger lifetime τ_Aug_ (green dash-dotted line) using established literature data for the radiative and Auger coefficients^9^. The resulting total lifetime τ_tot_ = 1/(1/τ_SRH_ + 1/τ_rad_ + 1/τ_Aug_) is shown in Fig. 4 as solid blue line.

In order to obtain the τ_QSSPC_(Δ*n*) curve shown as triangles in Fig. 4 from the measured Δ*V*_rf_ vs. suns measurement shown in Fig. 2, the only undefined parameter left is the mobility sum µ_B_, which according to literature^2,9,10^ should be in the range of 10–20 cm^2^/Vs. We hence vary µ_B_ over this range and compare the resulting τ_QSSPC_(Δ*n*) data with the calculated τ_tot_(Δ*n*) shown as solid blue line in Fig. 4. The best agreement is obtained for µ_B_ = 16.7 cm^2^/Vs, which we hence identify with the mobility sum of our evaporated MAPbI_3_ layer. This value lies well within the mobility range reported in the literature for this type of material^2,9,10^. It is obvious from the 1/Δ*n* dependence of the QSSPC lifetime in Fig. 4 that the QSSPC measurement is performed under such high illumination conditions, that radiative recombination is the limiting recombination process in the examined MAPbI_3_ layer.

**Figure 5:**
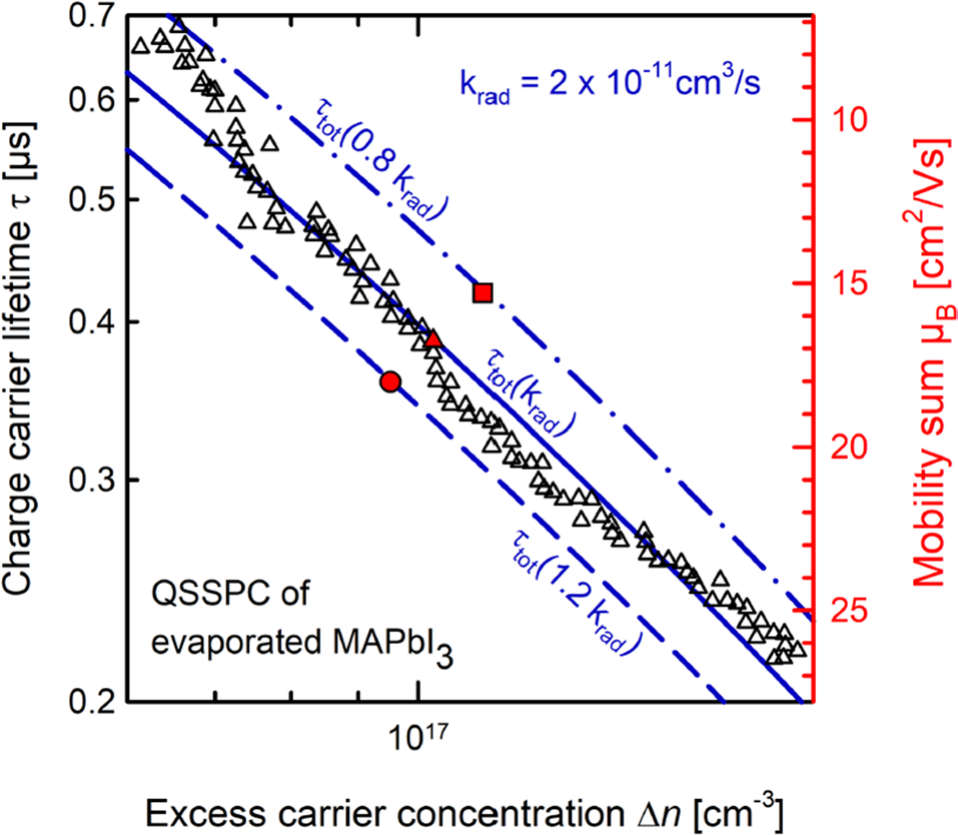
For the mobility uncertainty analysis, we consider the first standard deviation of k_rad_, which in the lower bound is 1.6 × 10^–11^ cm^3^/s (dashed-dotted blue line) and in the upper bound is 2.4 × 10^–11^ cm^3^/s (dashed blue line). For each bound, the best agreement of a representative datapoint of the τ_QSSPC_(Δn) measurement (red square and circle) with the calculated τ_tot_(Δn) curve yields an upper and lower limit of the mobility sum µ_B_ of 18.0 cm^2^/Vs and 15.3 cm^2^/Vs, respectively.

Figure 5 shows an uncertainty analysis of the impact of the assumed coefficient of radiative recombination *k*_rad_ on the extracted µ_B_ value. We assume two boundaries for *k*_rad_, the lower bound being *k*_rad_ = 1.6 × 10^–11^ cm^3^/s and the upper bound being *k*_rad_ = 2.4 × 10^–11^ cm^3^/s, both value originating from the first standard deviation in *k*_rad_ as determined in Ref.^9^. As the calculated *k*_rad_-dependent radiative lifetime is the dominant part of the total lifetime τ_tot_(Δ*n*) in the QSSPC measurement range, we observe a pronounced impact on the calculated τ_tot_(Δ*n*)-curve. For the lower bound of *k*_rad_, the total lifetime shifts to larger values (dashed-dotted blue line), while the upper bound of *k*_rad_ results in lower total lifetimes (dashed blue line). Our extraction of the mobility sum µ_B_ is based on the best agreement of the µ_B_-dependent τ_QSSPC_(Δ*n*) with the *k*_rad_-dependent τ_tot_(Δ*n*). In Fig. 5, the τ_QSSPC_(Δ*n*) curve is represented by one exemplary measurement point (red triangle), which perfectly overlaps with the τ_tot_(Δ*n*) curve for the mobility sum of µ_B_ = 16.7 cm^2^/Vs. For the lower bound (*k*_rad_ = 1.6 × 10^–11^ cm^3^/s), the best agreement of τ_QSSPC_(Δ*n*) (red square) with τ_tot_(Δ*n*) (dashed-dotted blue line) is obtained for a mobility sum of µ_B_ = 15.3 cm^2^/Vs. The best match of τ_QSSPC_(Δ*n*) (red dot) with τ_tot_(Δ*n*) (dashed blue line) for the upper bound (*k*_rad_ = 2.4 × 10^–11^ cm^3^/s) yields a mobility sum of µ_B_ = 18.0 cm^2^/Vs. This results in a total uncertainty range for µ_B_ of 15.3 cm^2^/Vs to 18.0 cm^2^/Vs. The QSSPC measurement is hence suited for extracting the mobility sum of electrons and holes in the perovskite layer with a reasonable level of accuracy.

From the lifetime measurements, one can now deduce a suns-implied open-circuit voltage characteristic of the examined perovskite layer. The implied voltage *V*_impl_ corresponds to the quasi-Fermi level splitting in the perovskite *V*_impl_ = (*E*_F*n*_ – *E*_F*p*_)/*q*, where *E*_F*n*_ and *E*_F*p*_ are the quasi-Fermi levels of electrons and holes. *V*_impl_ provides the highest possible *V*_oc_ as a function of illumination intensity in a solar cell fabricated on this material. As our examined MAPbI_3_ is undoped, the lifetime measurements are carried out under high-injection conditions. Therefore, the implied voltage can be calculated using the simple equation *V*_impl_ = 2*kT*/*q* × ln(Δ*n*/*n*_i_) with *k* being the Boltzmann constant, *T* the absolute temperature, and *n*_i_ = 8.04 × 10^4^ cm^−3^ the intrinsic carrier concentration of MAPbI_3_^2^.

**Figure 6:**
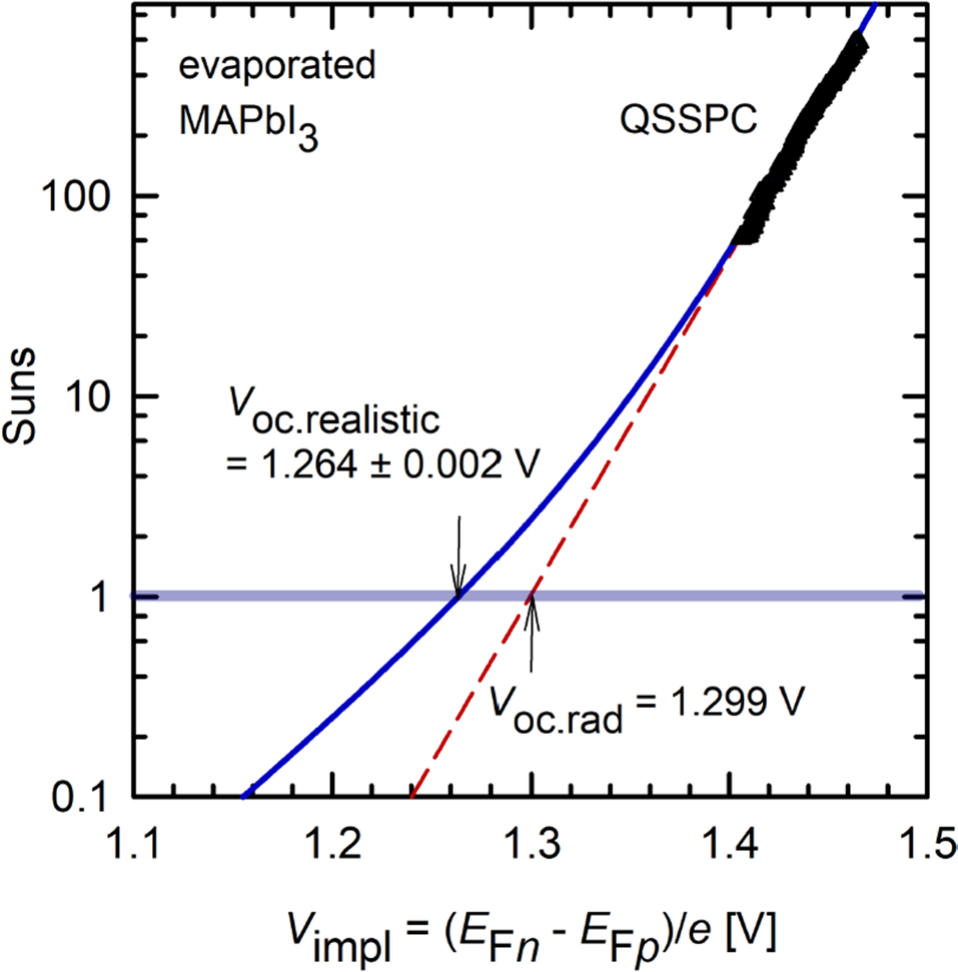
Suns-implied voltage characteristic of the examined MAPbI_3_ sample. The triangles are calculated from the QSSPC measurement shown in Fig. 4. The dashed red line is an extrapolation of the QSSPC data assuming only radiative recombination, i.e. is obtained from the τ_rad_ curve in Fig. 4. The solid blue line includes the SRH recombination and is obtained from the τ_tot_ curve shown in Fig. 4.

Figure 6 shows the resulting suns-*V*_impl_ characteristics of the examined MAPbI_3_ layer as calculated from the curves shown in Fig. 4. The symbols shown are directly calculated from the QSSPC measurement shown in Fig. 4. The QSSPC measurement is fully dominated by radiative recombination, since it is performed at very high injection levels. The dashed red line is obtained by assuming that only radiative recombination takes place, i.e. it is calculated from the τ_rad_ curve in Fig. 4. Extrapolation of the red dashed curve to one sun provides us with a *V*_oc.rad_-limit of 1.299 V for the MAPbI_3_ layer in the radiative limit. Note that the *V*_oc.rad_ limit should mainly be equal for the same kind of material, as it does not dependent on any defect-related SRH recombination. In order to determine a more relevant one-sun *V*_oc_ for the particular sample including SRH recombination, we include the TRPL data measured at much lower injection densities. The TRPL lifetime is largely limited by the defect-related SRH recombination and is used to determine the total lifetime τ_tot_ in Fig. 4. Reversely calculating the total lifetime curve, including now SRH as well as radiative recombination into the suns-*V*_imp_ curve, results in the blue solid line in Fig. 6. This line provides the most realistic limit to what can be regarded as best-case scenario obtained in a solar cell made of the examined MAPbI_3_ layer. From the blue solid line, we extract a realistic one-sun *V*_oc_ limit of *V*_oc.realistic_ = 1.264 ± 0.002 V. We can hence conclude that the SRH recombination in our MAPbI_3_ layer reduces the maximally reachable *V*_oc_ by *V*_oc.rad_ – *V*_oc.realistic_ = 35 ± 2 mV. This reduction obtained from our analysis is an excellent measure for the quality of the MAPbI_3_ layer with respect to the deviation from the perfect defect-free material. The methodology presented here, based on combining QSSPC and TRPL measurements, is therefore suited for the detailed characterization of the potential of metal halide perovskite layers.

## Conclusions

We have demonstrated the feasibility of contactless quasi-steady-state photoconductance (QSSPC) measurements, as used before only for silicon wafer characterization, to thin layers of MAPbI_3_ perovskites. Through a calibration of the inductively coupled WCT-100 tool at ultralow conductances, we revealed a much higher sensitivity of 420 V/S at ultralow photoconductances prevalent in the MAPbI_3_ thin-film, which is one order of magnitude larger than the sensitivity in the conductance range where silicon samples are routinely measured. Hence, a sufficiently large signal-to-noise ratio was measured despite the ultralow photoconductance values in the very thin (500 nm) MAPbI_3_ layers. The QSSPC measurements were performed at relatively large illumination intensities (40–590 suns), where radiative recombination limits the total lifetime of the perovskite. Hence, using the radiative recombination coefficient of MAPbI_3_ reported in the literature, we were able to pinpoint the sum of electron and hole mobility to µ_B_ = 16.7 cm^2^/Vs within the examined perovskite layer. We have determined the µ_B_ uncertainty range assuming that the radiative recombination coefficient is known within an accuracy of ± 20%, which resulted in a µ_B_ uncertainty range of (15.3–18.0) cm^2^/Vs. Furthermore, we have deduced a suns-implied open circuit voltage characteristic of the examined perovskite layer from the lifetime measurements. By extrapolating to one sun, we extracted a radiative one-sun *V*_oc_-limit of *V*_oc.rad_ = 1.299 V. Taking an additional time-resolved PL measurement into account, performed at much lower injection densities where SRH dominates the total recombination, a realistic one-sun *V*_oc_ limit of *V*_oc.realistic_ = 1.264 ± 0.002 V was determined. The difference *V*_oc.rad_ – *V*_oc.realistic_ = 35 ± 2 mV is a good measure to assess the quality of the MAPbI_3_ material and should be minimized.

### Supplementary Information

Supplementary Information.

## Data Availability

The datasets generated during and analyzed during the current study are available from the corresponding author on reasonable request.
