# Author Correction: Contactless quasi-steady-state photoconductance (QSSPC) characterization of metal halide perovskite thin films

**DOI:** 10.1038/s41598-024-70190-3

**Published:** 2024-08-19

**Authors:** Benjamin Grimm, Sascha J. Wolter, Jan Schmidt

**Affiliations:** 1 Institute for Solar Energy Research Hamelin (ISFH), Am Ohrberg 1, 31860 Emmerthal, Germany; 2 Department of Solar Energy, Institute of Solid‑State Physics, Leibniz University Hannover, Appelstr. 2, 30167 Hannover, Germany

Correction to: *Scientific Reports* 10.1038/s41598-023-37745-2, published online 10 July 2023

The original version of this Article contains an error in the Introduction section.

“In this paper, we employ the contactless quasi-steady-state (QSSPC) method, a measurement technique developed to measure the injection dependent lifetime in silicon by inductively coupling an illuminated sample to an rf bridge while simultaneously recording the time-dependent illumination intensity [zitat sinton].”

now reads:

“In this paper, we employ the contactless quasi-steady-state (QSSPC) method, a measurement technique developed to measure the injection-dependent lifetime in silicon by inductively coupling an illuminated sample to an rf bridge while simultaneously recording the time-dependent illumination intensity^6^.”

The original Article has been corrected.

