# Supplementary material for: Contactless quasi-steady-state photoconductance (QSSPC) characterization of metal halide perovskite thin films
Source: Sci Rep. 2023 Jul 10;13:11163. doi: 10.1038/s41598-023-37745-2 (PMC10333188; doi:10.1038/s41598-023-37745-2)
Supplement: Supplementary file 1 — Supplementary Information. [file 41598_2023_37745_MOESM1_ESM.pdf]

Supplemental material for

Contactless Quasi-Steady-State Photoconductance (QSSPC) Characterization of Metal Halide Perovskite Thin Films

Benjamin Grimm,[1,2,*] Sascha J. Wolter,[1] and Jan Schmidt[1,2]
[1]*Institute for Solar Energy Research Hamelin (ISFH), Am Ohrberg 1, 31860 Emmerthal, Germany*
[2]*Department of Solar Energy, Institute of Solid-State Physics, Leibniz University Hannover, Appelstr. 2, 30167 Hannover, Germany*
*Corresponding author, Email: b.grimm@isfh.de

A. Coevaporation of $MAPbI_3$ thin film

500 nm thick methylammonium lead iodide ($MAPbI_3$) perovskite layers were deposited on 2.5 x 2.5 cm^2 borofloat glass substrates. Lead iodide (PbI_2) and methylammonium iodide (MAI) were thermally co-evaporated in a lab-type evaporation chamber (Mini SPECTROS, Kurt J. Lesker Company) inside a glovebox with nitrogen atmosphere. The borofloat glass substrates were initially cleaned in an ultrasound bath in three consecutive steps using mucasol, isopropanol and acetone. They were then rinsed with water to remove any remaining soap residues.

Subsequently, the substrates were placed in a substrate holder, where the temperature was kept at 25 °C during the entire deposition process. After the chamber pressure was decreased to below 2 x 10^{-6} Torr the preheating started. First, the PbI_2 crucible was heated to 200°C. Reaching that temperature, the MAI crucible was heated to 100°C while the PbI_2 crucible was further heated to 300°C. After opening the crucible shutters and reaching a PbI_2 deposition rate of 1.08 Å/s as well as a MAI crucible temperature of 118°C, the co-evaporation process started with the subsequent opening of the substrate shutter. To ensure a laterally homogeneous deposition, the substrate holder was rotated at 20 rpm. Details of our process can be found in Refs. S1 and S2.

B. Time-Resolved Photoluminescence (TRPL)

TRPL measurements were performed using a FluoTime 300 tool from PicoQuant. The measurement is based on a 20 ps (FWHM) short-pulse laser illumination of the sample at 100 kHz with a 505 nm laser. It includes an ultra-precise detection of single photons via a TimeHarp 260 photon counter with a base resolution of 25 ps. By selecting a low excitation energy of 26 pJ/cm^2 we decrease the probability of a second undetectable photon in each 10 µs laser-pulse-cycle to below 1%. Thereby, only one, if any, photon is detected in each cycle. The emission time is tracked and the photon is counted in a histogram which consists of time bins with a width of 800 ps. Over many excitation-cycles, the histogram forms a decay curve. We then interpret the resulting decay curve as discussed in Ref. 3. The dynamic nature of the TRPL does not allow to measure the actual excess carrier lifetime during the excess carrier decay. However, we have estimated the excess carrier concentration to be much lower ($\leq 10^{15}$ cm^{-3}) in our $MAPbI_3$ film compared to the QSSPC measurement. Figures S1 shows an exemplary measurement of the measured PL

signal $PL^{1/2}$ as a function of time after the laser excitation pulse. The lifetime approaches in the asymptotic limit a constant value of τ_{TRPL} = 3 µs, which we identify with the Shockley-Read-Hall lifetime τ_{SRH}.

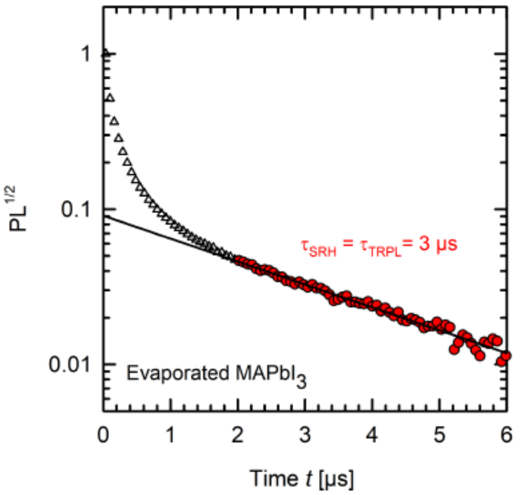

Figure S1: Exemplary time-resolved photoluminescence (TRPL) measurement of a 500 nm thick $MAPbI_3$ layer on borofloat glass using a Fluotime 300 spectrometer by PicoQuant. For t > 2 µs the PL decay curve becomes asymptotic and results in a lifetime of τ_{TRPL} = 3 µs.

The TRPL measurement depicted in Figure S1 features an initial multiexponential decay. For longer times of t > 2 µs the asymptotic decay can be fitted monoexponentially with a decay time of τ_{SRH} = τ_{TRPL} = 3 µs which indicates a high electronic thin-film quality.

C. X-Ray Diffraction (XRD)

XRD measurements were performed with a Malvern Panalytical Empyrean XRD tool using CuK_α radiation (λ = 1.54 Å).

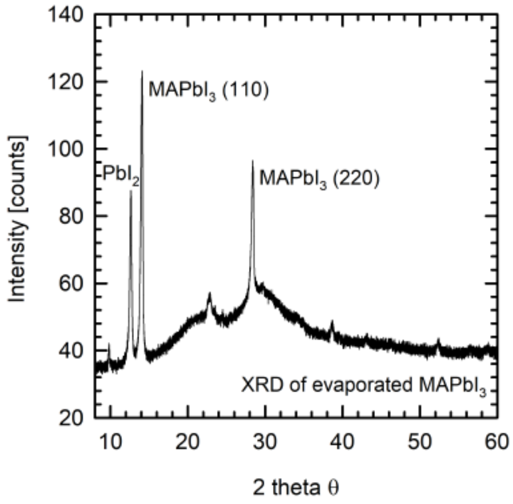

Figure S2: XRD measurement of evaporated $MAPbI_3$ thin film with a Malvern Panalytical Emyrean XRD tool.

The XRD measurement shown in Figure S2 depicts two clear peaks associated with the pure tetragonal (β) phase of $MAPbI_3$. The larger Peak at 2θ of 14.07° is associated with the $MAPbI_3$ (110) lattice orientation

while the smaller peak at 2θ of 28.04° represents the second-order reflex of $MAPbI_3$ at (220) lattice orientation [S3, S4]. Another peak at 2θ of 12.6° indicates the presence of PbI_2. Figure S2 clearly shows that the tetragonal $MAPbI_3$ phase is the dominant phase in our films, but PbI_2 is also present.

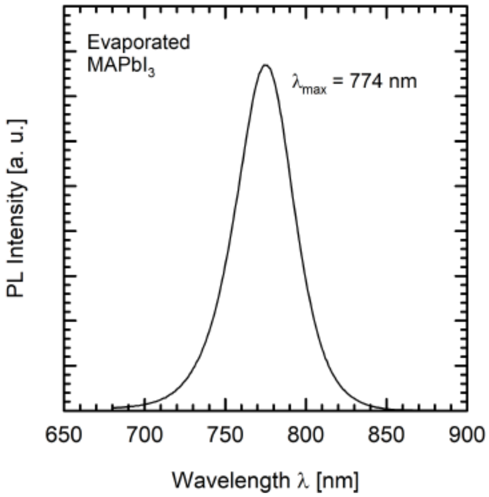

Figure S3: Photoluminescence measurement of a 500 nm $MAPbI_3$ thin film on borofloat glass using a Fluotime 300 spectrometer by PicoQuant.

D. Spectral photoluminescence

For spectrally resolved measurements of the photoluminescence (PL) signal of the $MAPbI_3$ layer, we use a Fluotime 300 spectrometer by PicoQuant. Figure S3 shows a spectral measurement of the emitted PL intensity, resulting in a well-shaped PL peak with a peak wavelength of 774 nm, corresponding to a bandgap of 1.6 eV, as typical for high-quality tetragonal $MAPbI_3$ at room temperature [S5,S6].

[S1] Wolter, S. J. Determination and influence evaluation of the acoustic impedance ratio for thermal co-evaporation. *Appl. Phys. Lett.* **113**, 013301; DOI: https://doi.org/10.1063/1.5037403 (2018).

[S2] Niepelt, R. Application of experimentally determined acoustic impedance ratio for homogeneous co-evaporation of perovskite absorbers. *IEEE 7th world conference on photovoltaic energy conversion (WCPEC)* 508-511 (2018).

[S3] Basumatary, P. and Agarwal, P. Photocurrent transient measurements in MAPbI3 thin films. *Journal of Materials Science: Materials in Electronics* **13**, 10047, DOI: https://doi.org/10.1007/s10854-020-03549-7 (2020)

[S4] Guo, X., McCleese, C., Kolodziej, C., Samia, A. C. S., Zhao, Y. and Burda, C. Identification and characterization of the intermediate phase in hybrid organic-inorganic $MAPbI_3$ perovskite. *Dalton Trans* **45**, 3806, DOI: 10.1039/c5dt04420k (2016)

[S5] Kong, W., Ye, Z., Qi, Z., Zhang, B., Wang, M., Rahimi-Iman, A. and Wu, H. Characterization of an abnormal photoluminescence behavior upon crystal-phase transition of perovskite $CH_3NH_3PbI_3$. *Phys. Chem. Chem. Phys.* **17**, 16405, DOI: 10.1039/c5cp02605a (2015).

[S6] Wehrenfennig, C. Liu. M., Snaith, H. J., Johnston, M. B. and Herz, L. Homogeneous Emission Line Broadening in the Organo Lead Halide Perovskite $CH_3NH_3PbI_{3-x}Cl_x$. *J. Phys. Chem. Lett* **5**, 1300, DOI: dx.doi.org/10.1021/jz500434p (2014).
